# Rare Cause of Late Recurrent Angina following Coronary Artery Bypass Grafting: Iatrogenic Aortocoronary Arteriovenous Fistula Causing Coronary Steal

**DOI:** 10.1155/2018/6913737

**Published:** 2018-06-24

**Authors:** Jayakumar Sreenivasan, Muhammad Ayub, Neha Yadav, Yasmeen Golzar

**Affiliations:** ^1^Department of Internal Medicine, John H. Stroger Hospital of Cook County, Chicago, IL 60612, USA; ^2^Department of Internal Medicine, Division of Cardiology, John H. Stroger Hospital of Cook County, Chicago, IL 60612, USA

## Abstract

Iatrogenic aortocoronary arteriovenous fistula is a very rare complication of coronary artery bypass grafting in which one of the arterial grafts inadvertently forms a fistulous tract with a cardiac vein, shunting blood from the anastomosed coronary artery. We report a patient with an iatrogenic left internal mammary artery graft to cardiac vein fistula presenting with recurrent angina three years after a three-vessel coronary artery bypass grafting.

## 1. Introduction

This case of iatrogenic aortocoronary arteriovenous fistula (ACAVF) is interesting because it is a rare but important complication of coronary artery bypass grafting (CABG) which can result in significant clinical consequences like heart failure, tamponade, and death. To the best of our knowledge, only 38 similar cases of ACAVF have been reported in the literature. We have included a brief review of the literature on this rare complication and the various available treatment options.

## 2. Case Presentation

A 61-year-old woman with diabetes mellitus, hypertension, and hyperlipidemia initially presented with unstable angina; a regadenoson stress nuclear myocardial perfusion imaging (MPI) revealed anterolateral wall ischemia. Subsequent coronary angiography demonstrated severe stenoses of the left anterior descending (LAD) artery, left circumflex artery, and right coronary artery. She underwent CABG with a left internal mammary artery (LIMA) graft to the LAD artery and saphenous venous grafts (SVG) to the right posterior descending (RPDA) and obtuse marginal 4 (OM4) arteries. After CABG, the patient was doing well on guideline-directed medical therapy. Three years later, she presented with acute onset of chest pain suggestive of unstable angina. On admission, her heart rate was 80/min, blood pressure was 140/86 mmHg, and her physical exam was unremarkable.

## 3. Investigations

**Figure 1:**
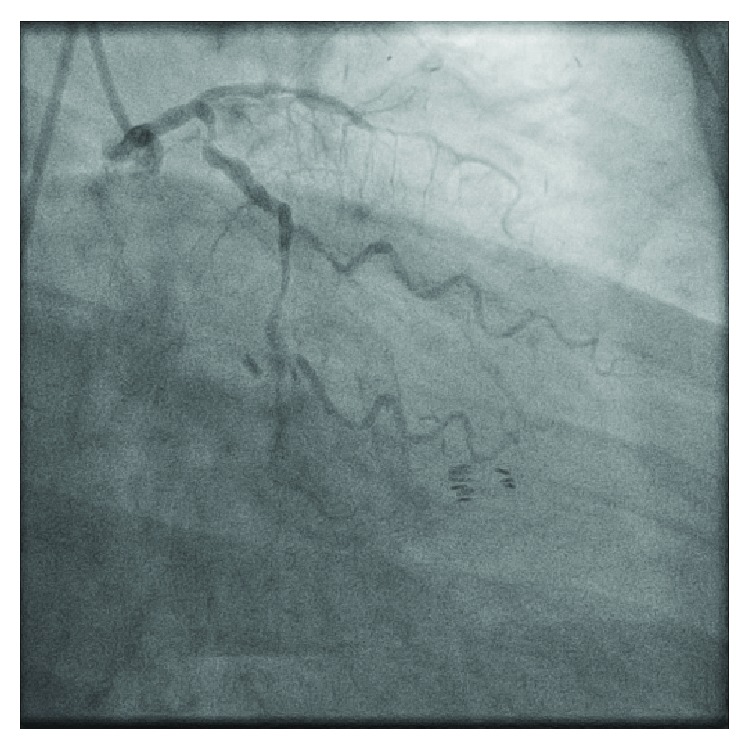
Right anterior oblique (RAO) caudal view of the left coronary artery with severe disease of the left anterior descending (LAD) artery.

**Figure 2:**
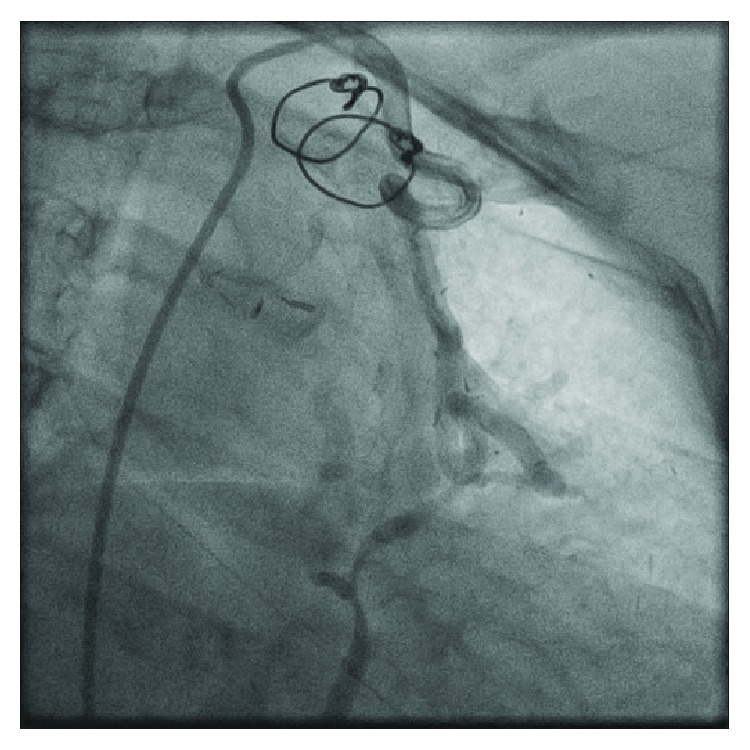
Graft angiography demonstrated a tortuous left internal mammary artery (LIMA) graft with flow going to the coronary sinus forming an aortocoronary arteriovenous fistula.

**Figure 3:**
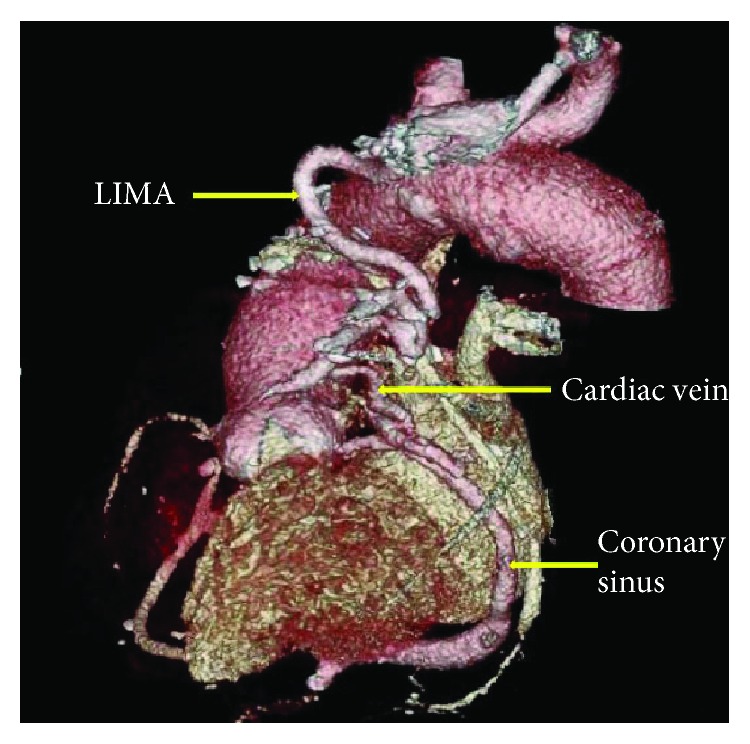
Cardiac computed tomography (CT) angiography with 3-dimensional reconstruction showed distal left internal mammary artery (LIMA) to left anterior descending (LAD) bypass artery communicating with a cardiac vein, which is eventually communicating with the coronary sinus, suggesting a coronary arteriovenous fistula.

Electrocardiogram showed normal sinus rhythm without any evidence of ischemia. Serially monitored cardiac enzymes were within normal limits, and transthoracic echocardiography revealed normal ejection fraction with no distinct regional wall motion abnormalities. Regadenoson stress nuclear MPI showed anterolateral ischemia, a territory which should have been supplied by the LIMA to LAD graft. Diagnostic coronary angiography revealed known severe native, three-vessel coronary artery disease. The native LAD vessel was very small. Proximal LAD was medium sized. Mid LAD had 100% stenosis at the origin of diagonal branch. Distal LAD was not visualized (Figure 1 and Video 1). Graft angiography was significant for a very large tortuous LIMA graft with poor visualization of the LAD artery after touchdown (Figure 2 and Video 2). Upon contrast injection of the LIMA graft, there was immediate opacification of the coronary sinus suggesting a fistulous tract between the LIMA graft and a cardiac vein (Figure 2 and Video 1). SVG grafts to the RPDA and OM4 arteries were patent. Cardiac computed tomographic angiography (CTA) confirmed the cardiac catheterization findings of a LIMA graft to coronary sinus fistula (Figure 3).

## 4. Clinical Decision Making, Treatment, Outcome, and Follow-Up

The case was discussed with the cardiac surgical team as well as in a multicenter interventional conference to explore both surgical and percutaneous options. A percutaneous approach of coiling of the fistula is anatomically complex; a surgical approach which would involve ligation of ACAVF and redo CABG, would put the patient at higher risk of perioperative cardiac morbidity and mortality. Because of the coronary arterio-venous fistula with steal, it was unclear whether revascularization of the native LAD would be of any significant benefit to the patient. After full discussion about the risks versus benefits of treatment options with the heart team, the patient elected for medical management and intervention only if she has escalating symptoms. The patient's anti-anginal therapy was optimized with Metoprolol 50 mg daily, which she tolerated well with gradual improvement in her exercise tolerance and resolution of her anginal symptoms. The patient is currently asymptomatic at 1 year follow-up. Repeat cardiac stress testing may be pursued if she has recurrent symptoms. The tentative plan is to offer her interventional coil closure or surgical closure of the ACAVF if she has recurrent angina or heart failure symptoms.

## 5. Discussion

Iatrogenic ACAVF is a rare complication of CABG causing significant morbidity. To the best of our knowledge, only 38 cases have been reported [1]. ACAVF can become symptomatic over the course of time. The most common presentation is angina due to coronary steal phenomenon involving the affected coronary artery [2]. Other less commonly reported potential outcomes are high output heart failure as a sequela of chronic arteriovenous fistula and rupture leading to cardiac tamponade [2–4]. The LAD is the most commonly involved artery, representing 60% of cases of iatrogenic ACAVF following CABG [2]. Possible risk factors for development of post-CABG iatrogenic ACAVF are myocardial fibrosis from prior infarction or cardiac surgery as well as a LAD artery deeply embedded in thick epicardial fat which makes it surgically challenging to separate it from the adjacent great cardiac vein [2, 3]. Cardiac computed tomography (CT) or magnetic resonance angiography (MRA) are the best available noninvasive imaging modalities for visualization of ACAVF [5, 6]. Cardiac catheterization with graft angiography is the best testing modality for the diagnosis of ACAVF. The appearance on angiography is a very large caliber LIMA which has undergone hypermaturation due to it being connected to a low-pressure venous system. As of now, there are no robust evidence-based data to help guide treatment for patients with ACAVF. Management of ACAVF depends on the presentation and symptomatology of the patients. Spontaneous closure of ACAVF in two asymptomatic patients has been reported so far [7]. Hence, conservative therapy is preferred for asymptomatic patients and patients with a good response to optimal medical treatment of angina as like in our case [2]. Our case is the third in literature for conservative medical management of ACAVF. We think medical management of ACAVF should include anti-anginal therapy particularly, a beta blocker in line with medical therapy for stable coronary artery disease until we have further prospective investigations for this unique clinical scenario. Patients who are persistently symptomatic due to ACAVF despite optimal medical therapy have primarily been treated with surgical ligation of the fistula and bypassing the diseased coronary artery [3]. With current advancements in innovative percutaneous techniques, coil or balloon embolization of the fistula and stenting of the unbypassed artery are the promising alternative treatment options over surgical approaches [8–13].

## Supplementary Materials

Supplementary 1 Video 1: left coronary angiography showing medium-sized proximal LAD with 100% stenosis at the origin of the first diagonal branch and poorly visualized distal LAD. First diagonal and left circumflex artery had severe diffuse atherosclerosis.

Supplementary 2 Video 2: graft angiography revealing a very large-sized, tortuous left internal mammary artery (LIMA) graft with flow going to the coronary sinus from a coronary arteriovenous fistula and poor visualization of left anterior descending (LAD) artery after touchdown.
